# Assessing preferences for adult versus juvenile features in young animals: Newly hatched chicks spontaneously approach red and large stimuli

**DOI:** 10.3758/s13420-024-00638-z

**Published:** 2024-08-16

**Authors:** Laura V. Freeland, Michael G. Emmerson, Vera Vasas, Josephine Gomes, Elisabetta Versace

**Affiliations:** School of Biological and Behavioural Sciences, Queen Mary University of London, London, UK

**Keywords:** Predispositions, Social behaviour, Chicks, Adult, Juvenile, Gallus gallus

## Abstract

Young precocial birds benefit from staying close to both their mother and their siblings, while prioritising adults, which provide better care. Which features of the stimuli are used by young birds to prioritise approach and eventually attachment to adults over siblings is unknown. We started to address this question in newly hatched domestic chicks (*Gallus gallus*), focusing on their spontaneous preferences for visual features that systematically vary between adult and juvenile chickens, and that had previously been identified as attractive: size (larger in adults than in juveniles) and colour (darker and redder in adults than in juveniles). Overall, chicks at their first visual experience, that had never seen a conspecific beforehand, were most attracted to the red and large stimuli (two adult features) and spent more time in close proximity with red stimuli than with yellow stimuli. When tested with red large versus small objects (Exp. 1), chicks preferred the large shape. When tested with yellow large and small objects (Exp. 2), chicks did not show a preference. Chicks had a stronger preference for large red stimuli (vs. small yellow objects) than for small red stimuli (vs. a large yellow object) (Exp. 3). These results suggest that the combination of size and colour form the predisposition that helps chicks to spontaneously discriminate between adult and juvenile features from the first stages of life, in the absence of previous experience, exhibiting a preference to approach stimuli with features associated with the presence of adult conspecifics.

**Supplementary Information:**

The online version contains supplementary material available at 10.3758/s13420-024-00638-z.

## Introduction

**Fig. 1:**
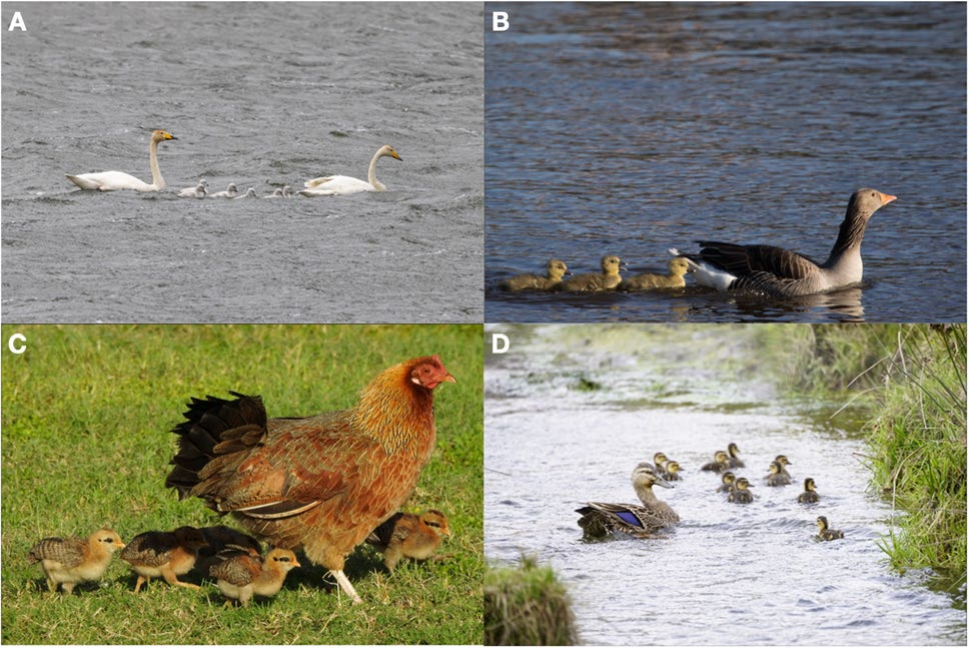
In precocial birds, juveniles imprint on and follow their mother soon after hatching. (**A**) Whooper swan (*Cygnus cygnus*), image credit: Mason Flint, Macaulay Library ML505596021. (**B**) Graylag goose (*Anser anser*), image credit: Roland Pfeiffer, Macaulay Library ML442529161. (**C**) Domestic chicken (*Gallus gallus*), image credit: Joel Gilb, Macaulay Library ML198044131. (**D**) Mallard (*Anas platyrhynchos*), image credit: Ulises Cabrera Miranda, Macaulay Library ML494249871

To benefit from protection and acquire relevant information, young social animals should preferentially direct their attention towards adult conspecifics. Indeed, in many species we observe brood movements led by the mother, with juveniles following the adult (Fig. 1). In chickens, mother hens direct the behaviour of their chicks and buffer their responses to stressors (Edgar et al., 2016), with maternal contact extending up to 3 months, while chicks also remain in contact with their siblings (McBride et al., 1969). To help group cohesion, chickens and other precocial birds have evolved a fast-learning mechanism called *imprinting* (Bolhuis, 1991, 1999; McCabe, 2019; Vallortigara & Versace, 2018), which enables them to quickly learn the specific features of social partners and stay close to them. After a brief imprinting exposure with the mother hen (in experimental settings also with another natural or artificial moving object such as the ethologist Konrad Lorenz (1937), a plastic cylinder (Szabó et al., 2021; Vallortigara & Andrew, 1994; Versace et al., 2006) or even a shape displayed on a computer monitor (Lemaire et al., 2021a, 2021b; Versace et al., 2017a, 2017b; Wood, 2015), chicks develop attachment for the imprinting object, follow it when moves, and produce distress calls when they are separated from it. However, while young birds are exposed to the hen, they are often exposed to the siblings of the same batch too. In wild fowls, batches consist of four to six eggs (Ali et al., 2016; Nicol, 2015), which hatch at around the same time. This produces an interference between filial imprinting directed toward the mother hen, and other salient conspecifics. Interestingly, young chicks do imprint on their siblings, an adaptive behaviour that supports group cohesion and predator avoidance (P. Bateson, 1982; Lickliter & Gottlieb, 1988; Rivera et al., 2018; Vallortigara & Andrew, 1991; Zajonc et al., 1975). Despite these benefits, it would be maladaptive for chicks to preferentially imprint on their siblings, because the mother hen can provide more warmth, protection from predators, and relevant information compared to the inexperienced siblings. What mechanisms enable young precocial birds to preferentially orient to and then imprint on adult hens versus young siblings are unknown. This work focuses on visual features.

We hypothesise that spontaneous preferences available in the absence of previous experience – *predispositions* (Rosa-Salva et al., 2021; Versace et al., 2018, 2019; Versace & Vallortigara, 2015) – might help chicks to preferentially orient towards adult versus juvenile animals, and therefore to preferentially imprint on adults. We start addressing this question in newly hatched domestic chicks (*Gallus gallus*), focusing on their spontaneous visual preferences for cues associated with adults in terms of size and colour. We investigated visual stimuli because extensive evidence shows that newly hatched chicks primarily rely on vision (Bolhuis, 1999; Versace et al., 2017a, 2017b). At the beginning of life, chicks use low-level visual features to orient their initial approach preferences (reviewed in Rosa-Salva et al., 2021; Versace & Vallortigara, 2015; Versace et al., 2018). These predispositions include spontaneous preferences for the neck and head region (Johnson & Horn, 1988; Rosa-Salva et al., 2010, 2019; Versace et al., 2017a, 2017b), hollow objects (Versace et al., 2016), biological motion (Vallortigara et al., 2005), upward movement (Bliss et al., 2023), changes in speed (Rosa-Salva et al., 2016; Versace et al., 2019), combination of colour and biological motion (Miura & Matsushima, 2016), size (Rugani et al., 2010; Schulman et al., 1970) and colour (e.g., Miura & Matsushima, 2016; reviewed in Rosa Salva et al., 2015). It is not known whether predispositions are preferentially directed to adult-like features.

Regarding size, we reasoned that adult chickens are consistently larger than juveniles (Fig. 1), as in other vertebrate species. Accordingly, we hypothesized that the larger size may be a cue used by young animals to preferentially attach to their parents. In line with this idea, newly hatched chicks have been shown to react more to multicoloured balls with a diameter of 10–20 cm, being less responsive to diameters of 5 or 25 + cm (Schulman et al., 1970). This pattern is suggestive of a stronger preference for objects with a size similar to that of the mother hen versus objects with a size similar to that of newly hatched chicks. However, this work did not control for numerosity or for the relative preference for the same proximal stimulation (e.g., same extent of area, perimeter) distributed between larger versus smaller individuals. More recently, chicks previously imprinted have been tested for their preferences for objects of different size, colour and shape after imprinting (Rugani et al., 2010): when exposed to sets of identical objects, chicks chose on the basis of the larger quantity of objects in a set. When faced with objects of similar spatial extent (Exp. 3), chicks preferred the largest individual when objects were different from one another. Chicks preferred larger numerosities of similarly sized objects irrespective of the number of stimuli they had been imprinted on, choosing larger volumes rather than familiar numerosity when continuous features were controlled for. This work left open the question of the spontaneous preferences of chicks for objects that differ in colour and size, before they have been imprinted. Our experiments address this knowledge gap, focusing on the spontaneous approach responses for a single large versus five small objects (controlled for colour area), before imprinting takes place. We predicted that chicks should have a spontaneous preference for the single large object.

**Fig. 2:**
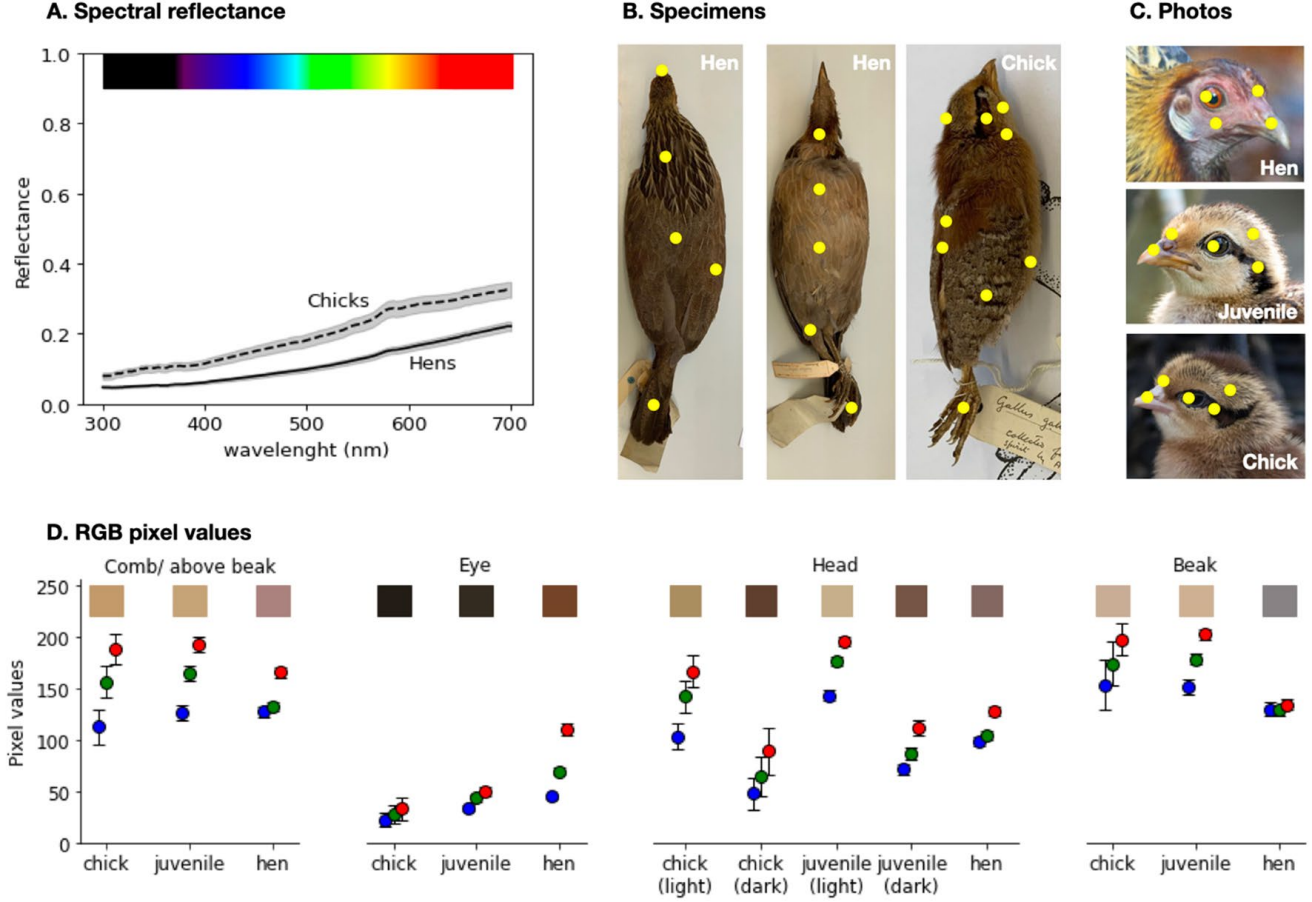
Colours of the red junglefowl (*Gallus gallus spadiceus*) hen and chicks. (**A**) The average spectral reflectance of the feathers of hens and chicks. The shadow indicates the standard error at each wavelength. (**B**) Example specimens of a hen and a chick of the red junglefowl. Yellow dots mark the body parts sampled with spectrophotometry. From the hens, we sampled the chest, neck underside, belly, upper leg, feet, tail underside, back, tail, neck from above, wing and beak areas. From the chicks, we took samples from the centre, side and underside of the head, from the underside of the neck, belly, feet, wing and from the dark and light stripes on the back. (**C**) Examples photos of the red junglefowls. Yellow dots mark the body parts sampled from photos: the comb (when present) or the area above the beak, the eye, the head (two measurements for the bicoloured chicks and juveniles) and the beak. Image credits: James Hully, Macaulay Library ML471555411, Kiri Zhang, Macaulay Library ML478986371, Andreas Boe, Macaulay Library ML200531451. (**D**) Mean ± SEM of RGB values of the pixels taken from photos of red junglefowl hens, juveniles and chicks. Red, green and blue dots indicate the red, green and blue components of the RGB values; the coloured rectangles illustrate the average colour

We also analysed colour preferences, another feature that can be used by chicks to discriminate between adults and juveniles. Previous work has shown that colour is central for behavioural choices in young chicks: for instance, colour is more important than shape in establishing imprinting preferences (Figler et al., 1974; Lemaire et al., 2021a, 2021b; Maekawa et al., 2006). Chicks are able to process fine differences in colours from the first days of life, as shown in pecking/foraging tasks (Ham & Osorio, 2007; Jones et al., 2001; Osorio et al., 1999). Interestingly, previous experiments (Miura et al., 2019) showed that red colour paired with biological motion is more effective than yellow colour matched with biological motion in eliciting chicks’ preferences. To clarify whether colour preferences reflect systematic differences between hens and juveniles in the wild, we investigated the spectral reflectance of the red junglefowl (*Gallus gallus*)*,* the closest living relative of the ancestral wild chicken before domestication (M.-S. Wang et al., 2020). We investigated systematic colour differences in feather colour and head colour between adults and juveniles using spectral reflectance measurements of stuffed bird skins from the Natural History Museum collections and pictures from the Macaulay library (see Fig. 2). Previous studies have shown that the head is a crucial body region for driving preferential approach choices (Johnson & Horn, 1988; Kobylkov & Vallortigara, 2024; Rosa-Salva et al., 2010, 2019; Zuk et al., 1992), with neurons sensitive to face-like patterns (Kobylkov et al., 2024), hence our focus on this area. As detailed below, we found that both hens and chicks display yellowish (see also McGraw et al., 2004) and brownish colours, but hens are darker (Fig. 2A). Moreover, in hens the head region is more reddish than in chicks and juveniles (Fig. 2D). Based on these observations, we predicted that young chicks should spontaneously prefer darker and more reddish colours, as a strategy to preferentially approach adult hens.

To test our predictions, we hatched chicks in darkness, and then presented them with stimuli of different size and colour using computer animations (Lemaire et al., 2021a, 2021b; see also Versace et al., 2017a, 2017b). At their first visual experience, chicks were given a two-choice test between one large shape versus five small shapes with the same total area. The colours differed between experiments. In Experiment 1, all shapes were red, hence the large stimulus had two “adult-like” features, i.e., size and colour. In Experiment 2, all shapes were bright yellow, hence the large stimulus has only one “adult-like” feature, i.e., large size. If chicks use size and colour to preferentially orient towards adults, we expected a stronger preference for the larger stimuli in both experiments, with a stronger effect in Experiment 1 compared to Experiment 2, due to the presence of two adult features in Experiment 1. In Experiment 3 we compared the combined effect of colour and shape. In Experiment 3A, shapes were one large red versus five small yellow and in Experiment 3B one large yellow versus five small red. We expected a stronger preference for the large red shape (Exp. 3A) compared to the five small red shapes (Exp. 3B), if the combination of two adult-like features was driving chicks’ preferences.

## Methods

### Colour assessment of feathers and head in hens and chicks

Spectral reflectance measurements were taken at the Bird Collection of the Natural History Museum (Tring, UK) using an AvaSpec-2048 spectrophotometer (Avantes) with an AvaLight-DHS (Avantes) light source on stuffed bird specimens. We measured 11 different areas on four specimens of hens, and nine different areas on three specimens of young chicks of red junglefowl (*Gallus gallus spadiceus*) (see Fig. 2B). Comparing hens and chicks, we observed that the average reflectance curves have the same shape (Fig. 2A), with a gradual increase in reflectance with larger wavelengths (characteristic of pheomelanin), resulting in the yellowish-brown appearance of the specimens (Fig. 2B and C). The skin of chicks consistently reflects more light at every wavelength, appearing brighter than hens’ skin.

In stuffed specimens, the soft parts of the body, including the head region, dry out; for this reason, we could not assess their original colour, despite the relevance of the head region outlined above. To compare the colour of the head of chicks and hens, we used photos of wild red junglefowl taken from the Macaulay library. We extracted the RGB pixel values of 60 hens, 43 juveniles, and nine newly-hatched chicks from comparable locations on the head (see Fig. 2C). None of the colours were saturated, but the hens appear slightly reddish in the comb/beak, eye and head areas (as shown by higher red than blue/green RGB values). The chicks and the juveniles have two main colours in these areas: the comb/beak and the lighter feathers on the head are slightly yellowish (with higher red/green than blue RGB values) and brighter than in hens as shown by the higher values in RGB (supporting observations in McGraw et al., 2004); the eye is black (without the orange band present in hens) and there is a dark brown stripe on the head. The beak is light brown in the chicks/juveniles and a darker grey in hens. Overall, the head regions of hens are darker and more reddish than those of chicks.

### Predisposition experiments

#### Subjects and rearing conditions

After standard incubation in darkness (37.7 °C and 40–60% humidity), chicks hatched in individual compartments and were carried to the experimental room for testing in an opaque box. For incubation and hatching we used a FIEM incubator S140ADS and hatchery H316DS. We tested domestic chicks (*Gallus gallus*) of the Ross 309 strain: 50 chicks (24 females, 26 males) in Experiment 1 (red stimuli), 62 chicks (27 females, 35 males) in Experiment 2 (yellow stimuli), 58 chicks (30 females and 28 males) in Experiment 3a (red large and yellow small stimuli), and 55 chicks (28 females and 27 males) in Experiment 3b (yellow large and red small stimuli) (see Table 1 for details). Based on a power analysis conducted before the experiments, a sample size of 60 subjects that made a choice was required for a medium effect size (in line with literature in the field and our previous work on this breed (Bliss et al., 2023; Wang et al., 2024), for a biologically relevant effect) of d = 0.37 (alpha = 0.05, power = 0.8) (Faul et al., 2009). Chicks were tested within 14 h after hatching (Versace et al., 2019).

**Table 1:** Number of chicks used in the experiments

	Total collected	Low quality videos	Total chicks analysed	No choice
Exp. 1	59 (28**♀**, 31**♂**)	9 (4**♀**, 5**♂**)	50 (24**♀**, 26**♂**)	4 (1**♀**, 3**♂**)
Exp. 2	69 (28**♀**, 41**♂**)	7 (1**♀**, 6**♂**)	62 (27**♀**, 35**♂**)	22 (5**♀,** 17**♂**)
Exp. 3A	60 (31♀, 29♂)	2 (1**♀**, 1**♂**)	58 (30♀, 28♂)	0
Exp. 3B	60 (32**♀**, 28**♂**)	5 (2**♀**, 3**♂**)	55 (28♀, 27♂)	2 (0♀, 2♂)

#### Apparatus and stimuli

**Fig. 3:**
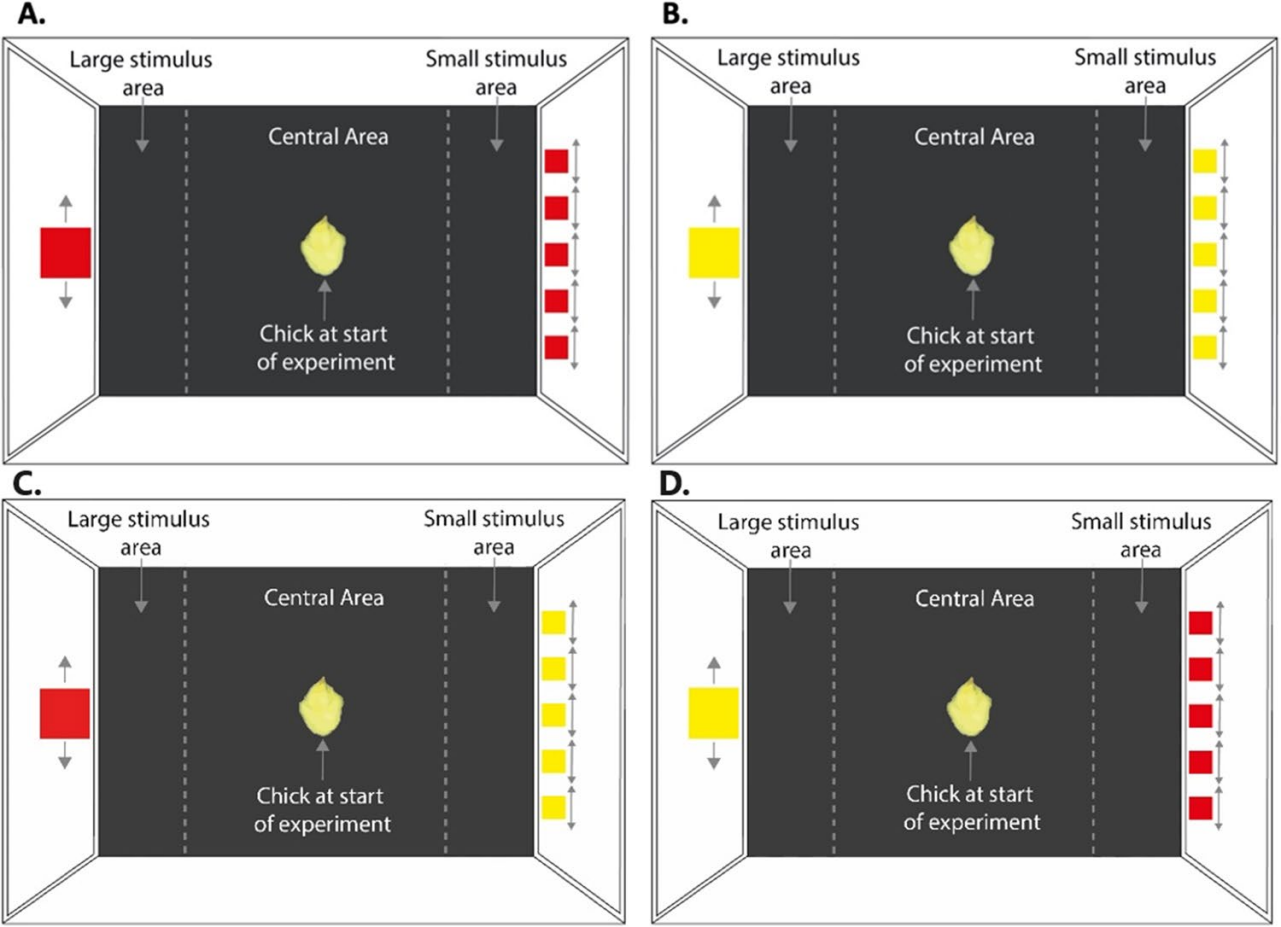
Experimental setup in Experiment 1 (**A**), Experiment 2 (**B**), Experiment 3A (**C**) and Experiment 3B (**D**). The arena was virtually divided into a central “no-choice” area where the chick was located at the beginning of the experiment, and two “side choice” areas. The centroid position of the chick was recorded and automatically tracked. In Experiment 1 (**A**), the chicks were given a choice between a large red square vs. five small red squares. To enhance approach responses, the stimuli moved horizontally, right and left, for an equal duration and distance on each side, as indicated by the arrows. In Experiment 2 (**B**), the chicks were given a choice between a large yellow square vs. five small yellow squares. In Experiment 3A (**C**), the chicks were given the choice between a large red square vs. five small yellow squares and in Experiment 3B (**D**) between a large yellow square vs. five small red squares

The experimental apparatus (Fig. 3) consisted of a wooden box (90 × 60 × 52 cm) with non-slip black floor, white walls, and one monitor at each end, displaying at 120 fps. The apparatus was divided into three virtual regions: the centre (54 × 60 cm) and two side choice areas (18 × 60 cm). A video camera located on top of the arena recorded the entire session. The experiments were recorded at 10 frames per second, at a resolution of 1,280 × 720 pixels.

The stimuli were displayed on two 24-in. Asus VG248QE monitors. A large shape (111.8 mm square and total area of 125 cm^2^) was shown on one monitor, and five small shapes (50 mm squares) on the opposite. The surface area of the single large shape was equal to the five small shapes. In Experiment 1, the stimuli were red (RGB: 255, 0, 0; Fig. 3A), in Experiment 2, yellow (RGB: 255, 255, 0; Fig. 3B), and in Experiment 3 yellow and red (Fig. 3C and D). We used the same colours and background used in the literature to be able to compare our results with the previous literature (Bliss et al., 2023; Wang et al., 2024). The white background facilitates the movement of chicks in a diurnal environment. We acknowledge that the technology of RGB monitors is just an approximation of colours experienced in nature. Further experiments should explore other colours as stimuli and background. To enhance approach responses, the stimuli moved horizontally (see Online Supplementary Material Videos 1 and 2). The larger stimulus moved 50 mm back and forth for 5 s while each smaller stimulus moved, one after the other, by 50 mm, so the objects moved for an equal duration and distance on each side. The stimuli remained still for a further 2 s, then a white screen was shown for 2 s. Following the white screen, the stimuli reappeared in a different position. Five different locations were played resulting in a total play-through of 60 ss. The right-left position of the stimuli in the arena was counterbalanced between subjects to remove any bias toward one side of the arena and to control for hemispatial dominance and bias (Daisley et al., 2008).

#### Procedure

At the start of the experiment, chicks were gently placed in the centre of the apparatus with the beak facing the long wall. In this way, through their lateral vision, subjects could see both stimuli at the same time, before deciding which stimuli to approach. Chicks were recorded for 20 min by a camera mounted above the centre of the arena. Previous studies observed predisposed behaviours in less than 20 min (Vallortigara et al., 2005); we ran a longer experiment as yellow stimuli have been previously found to elicit slower responses (Bateson & Jaeckel, 1976; Ham & Osorio, 2007; Taylor et al., 1969). Chicks were free to move in the apparatus. Chicks that did not move from the central area were excluded from preference analysis, in line with previous literature (Bliss et al., 2023; Vallortigara et al., 2005; Versace et al., 2016, 2024), because this did not provide any indication on their preferences. The number of chicks making a choice was used as an indication of attraction elicited by the stimuli.

#### Data analysis

The chicks’ position in the arena was tracked automatically using DeepLabCut, and then analysed in Python using standard modules (pandas, matplotlib, numpy, csv, os, scipy and statsmodel) (Mathis et al., 2018; *Python Software Foundation. Python Language Reference*, n.d.). Statistical analysis and data presentation were undertaken in R (version 4.3.2), using packages ggplot2, plyr, effsize (*R Core Team. R: A Language and Environment for Statistical Computing. R Foundation for Statistical Computing, Vienna, Austria*, 2023). For a recording to be included in the analysis, 90% of the video frames must have had an accuracy likelihood of 0.9 or greater (see Versace et al., 2016). In Experiment 2, in six videos the recording was not clear enough to identify the stimuli, and we discarded those data. See Table 1 for details.

To analyse the chicks’ initial preferences, we recorded the first choices, defined as the centroid of the chick entering either stimulus area. We scored a chick in an area based on the position of its centroid. Significant differences in the number of first choices for large versus small stimuli were assessed using binomial tests. We used a chi-squared test to determine the presence of sex differences in the number of chicks that did and did not approach the stimuli as previous studies have identified differences in reinstatement motivation between sexes (Lemaire et al., 2021a, 2021b; Pallante et al., 2021; Versace et al., 2017a, 2017b). The difference in latency to first approach (i.e., time elapsed until the chick made its first choice) was assessed using Mann–Whitney U tests, as latency did not match assumptions for parametric tests.

To analyse the chicks’ preference for the large stimulus, we calculated a preference index as:

$$Preference_{l\text{arg}e}=\frac{N_{frames\;at\;l\text{arg}e}}{N_{frames\;at\;l\text{arg}e}+N_{frames\;at\;small}}\times100$$

where 1 indicates a complete preference for the large stimulus, 0 a complete preference for the small stimuli, and 0.5 indicates no preference, in line with previous research (Lemaire et al., 2021a, 2021b; Versace et al., 2024).

Time was divided into 20 time bins of 1-min each in which the preference of each chick was calculated for each minute. As the preference index did not match assumptions for parametric tests, we used Friedman tests to determine if the chicks’ preference changed over time. The bins were then averaged over across each experiment to calculate the mean preference for the large stimulus. We used Wilcoxon tests (one-sample and independent samples, respectively) to establish if the preference for the large object deviated from chance level (0.5) and if the preference was different in the red (Exp. 1) versus yellow (Exp. 2) experiments.

To compare the rate of subjects that approached the stimuli between Experiments 1 and 2, we used a chi-squared test. To compare the responsiveness to the stimuli across experiments we analysed the total time spent in the centre using Mann–Whitney U tests. For all tests we set alpha to 0.05.

## Results

**Fig. 4:**
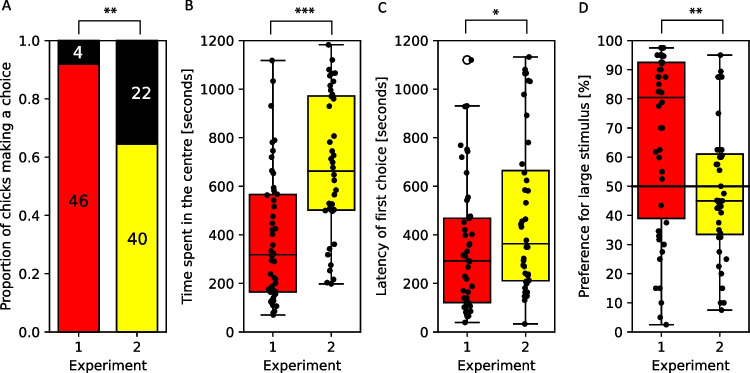
Comparisons of Experiment 1 (red stimulus) and Experiment 2 (yellow stimulus). The subplots show (**A**) the proportion of chicks that made any choice in the 20 min of the experiment and the number of chicks in each category; (**B**) the time the chicks spent in the centre area; (**C**) the time elapsed until they made their first approach to any of the stimulus areas; and (**D**) their preference for the large stimulus. In the subplots B–D, dots indicate the individual data points, and the boxplots show the median, first quartile to the third quartile of the data, the most extreme non-outlier points and the outliers. * *p* < 0.05, ** *p* < 0.01, *** *p* < 0.001

In Experiment 1, 46 out of 51 tested chicks (91%) approached at least one stimulus. We found no sex difference in the number of chicks that did/did not make a choice in Experiment 1 (χ^2^ = 0.134, *p* = 0.714). Chicks preferred to approach first the larger stimulus (35 vs. 11 chicks, 76%; *p* < 0.001). The preference for the large stimulus was different across time bins (χ^2^ = 56, *p* < 0.001). The difference was driven by the time it took the chicks to move towards their chosen stimulus and it disappeared after the first 5 min (bins 5–20: χ^2^ = 3.49, *p* = 0.479, W = 0.057). Out of the 20 1-min time bins, 17 showed significantly higher preference for the large stimulus (all except bins 1, 13 and 14; Fig. 3). As the preference remained at or above chance level (50%) throughout the test (Fig. 4), we collapsed the data across time bins. Overall, chicks preferred the single red large stimulus, as expected (Fig. 4D, mean ± SEM = 66.62 ± 5.18, median = 80.47; V = 840.5, *p* = 0.001, PS = 0.778).

**Fig. 5:**
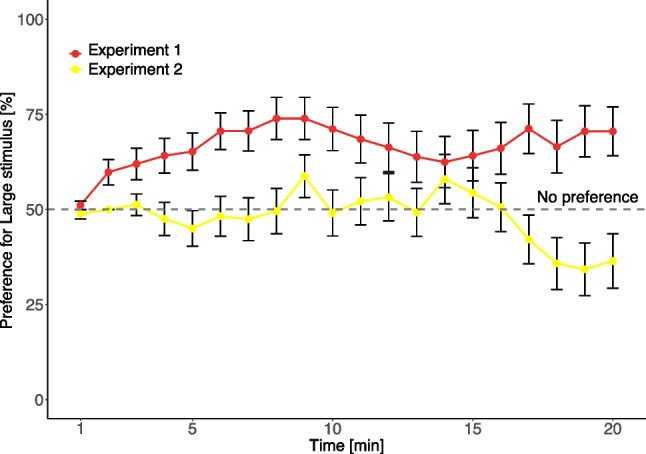
Preference for the large stimulus in Experiment 1 (red) and Experiment 2 (yellow), over time. Dots indicate the average preference of the chicks for that minute and error bars show the standard error of the mean

In Experiment 2, out of 62 tested chicks, 22 remained in the centre, and only 40 (65%) approached either of the experimental stimuli. Out of these, more chicks first approached the smaller stimuli than the large stimulus; however, the difference was not significant (24 vs. 16, 60%; *p* = 0.268). We found that females made a choice significantly more frequently than males (χ^2^ = 4.77, *p* = 0.029). The preference was not significantly different across time (χ^2^ = 8.6, *p* = 0.474, W = 0.0514). Looking at time bins individually (Fig. 4), two bins (18 and 19) reached the significance threshold (*p* = 0.035 and 0.024); however, neither was significant after correction for multiple comparisons (using Bonferroni correction, the overall alpha level of 0.05 would require a p value < 0.0025 for an individual hypothesis). As the preference remained between chance and preference for the small objects throughout the test (Fig. 5), we collapsed data across time bins. The preference for the large stimulus did not differ from chance level (Fig. 4D, mean ± SEM = 48.08 ± 6.14, median = 45.00; V = 365.5, *p* = 0.554, PS = 0.446). Overall, we did not find evidence for size preference when the stimuli were shown in yellow. However, we note the consistent trend towards the small yellow stimuli in the last four bins.

Comparing Experiments 1 and 2, we have found differences in the number of chicks that made a choice, and hence in the attraction of the stimuli. Chicks in Experiment 1 (red stimuli) were more likely to make a choice than in Experiment 2 (yellow stimuli) (Fig. 4A, χ^2^ = 10.56, *p* = 0.001). Chicks that approached the stimuli were significantly slower in Experiment 2 (yellow stimuli), (Fig. 4C, latency to first approach in Exp. 1, mean ± SEM: 345 s ± 39 s, latency in Exp. 2, mean ± SEM = 481 s ± 51 s; U = 677.0, *p* = 0.036) and accordingly spent more time in total in the centre area (Fig. 4B, red stimuli mean ± SEM = 389 ± 40 s, yellow stimuli mean ± SEM = 682 ± 46 s; U = 420, *p* < 0.001). We found a stronger preference for the large stimulus in Experiment 1 (red stimuli) versus Experiment 2 (yellow stimuli) (Fig. 4D, W = 1,269.5, *p* = 0.002, PS = 0.690). Taken together, these findings indicate a stronger responsiveness to large red than yellow stimuli against a white background.

**Fig. 6:**
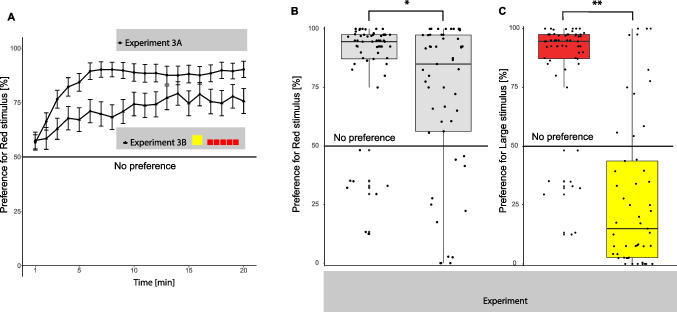
Preference over time for the red stimulus in Experiment 3A (large red and small yellow), shown by black circles, and Experiment 3B (large yellow and small red), shown by black triangles (**A**). The circles and triangles indicate the average preference of the chicks for that minute and error bars show the standard error of the mean. Preference for the red stimulus overall for Experiments 3A and 3B (**B**), and preference for the large stimulus in Experiments 3A and 3B (**C**). * *p* < 0.05, ** *p* < 0.01, *** *p* < 0.001

In Experiment 3, all chicks except two males made a choice, hence there was no significant difference between sexes in the number of chicks that did and did not make a choice in Experiment 3 (χ^2^ = 0.527, *p* = 0.468). In Experiment 3A (large red stimulus vs. five small yellow stimuli), overall chicks had a very strong preference for the large stimulus (Fig. 6A, mean ± SEM = 84.988 ± 0.903, median = 94.567, V = 1661, p < 0.001, PS = 0.971). In Experiment 3B (large yellow stimulus vs. five small red stimuli), overall chicks had a strong preference for the large stimulus (mean ± SEM = 27.545 ± 1.182, median = 15, V = 290, *p* < 0.001, PS = 0.203). The preference for red stimuli was significantly stronger with large red than with small red stimuli (W = 1905.5, *p* < 0.030, PS = 0.620), as predicted (Fig. 6B). The preference for the large stimuli was significantly stronger with the large red than with the large yellow stimuli (W = 2702.5, *p* < 0.01, PS = 0.880), as predicted (Fig. 6C).

## Discussion

It is not clear which visual features are used by young animals to approach and exhibit other affiliative responses toward adults over juveniles, despite the advantage for younger animals to receive parental care from adult animals (Edgar et al., 2016; McBride et al., 1969). In fowls, chicks spend most of the time with the mother during the first days, while a few weeks after hatching remain at times only with siblings (McBride et al., 1969). In dolphins, the initial play partner is the mother, before being replaced by other calves at a later stage of development (Mackey et al., 2014). In animals that benefit from remaining in close proximity with parents, such as poultry chicks, we expected to observe preferences for features associated with the presence of adults. It is not known whether and which visual features present in adults mediate these responses. Previous studies have shown preferences for features associated with the presence of adults (such as a larger shape) following exposure, rather than exploring the initial spontaneous preference that drives young to approach adults (Rugani et al., 2010), leaving the question open for initial spontaneous responses. We addressed this question by testing the spontaneous preferences of newly hatched domestic chicks (*Gallus gallus*). We investigated the attractiveness of size (large vs. small) and colour (red vs. yellow), two easily discernible features that differ between adults and juveniles. We hypothesised that by focusing on larger size and colours associated with adults, rather than on smaller size and colours associated with juveniles, chicks could reliably prioritise affiliative responses towards adults. We found that, in line with our hypothesis, chicks were more attracted to objects that show two features associated with adults (red colour and large size) than to objects that show one feature associated with adults and one associated with juveniles (red colour and small size). Because visual features spontaneously preferred by chicks, such as the preference for red and blue, enhance learning and generalisation (S. Wang et al., 2024), the spontaneous preferences for adults are likely to support faster learning of the feature associated with the mother hen, and in turn affiliative responses. Further experiments should evaluate the effect of cumulative adult features on imprinting efficacy.

In vertebrate species, adults grow larger than young animals, hence a larger size is a univocal feature associated with adulthood. Colour differs between species and developmental stages. To detect consistent colour differences between hens and chicks, we focused on the head region, which is important in driving chicks’ preferences (Johnson & Horn, 1988; Miura et al., 2019; Rosa-Salva et al., 2019; Versace et al., 2017a, 2017b). We analysed stuffed specimens and pictures of the red junglefowl (*Gallus gallus spadiceus*). The red junglefowl is the closest relative to the ancestral wild chickens before domestication (Wang et al., 2020). Overall, hens and chicks have similar spectral reflectance, but hens are darker and more reddish, compared to the lighter and more yellowish chicks. Based on this, we tested chicks for their spontaneous preference to approach red or yellow displays or large of small size, controlling for the area and movement of visual stimuli presented.

When presented with large versus small red visual stimuli (Exp. 1), visually inexperienced chicks had a spontaneous preference for the larger stimuli. This result is in line with previous findings for the most attractive size (Schulman et al., 1970) and for preference for larger objects (Rugani et al., 2010). This spontaneous preference for the larger stimulus overcame the chicks’ previously observed preference for larger numerosities (Rugani et al., 2010). In light of these results, it is possible to also reinterpret the results of Rugani et al. (2010), where chicks imprinted on a single distinct larger object (larger in volume, surface or perimeter) preferred larger objects, but chicks exposed to sets of identical objects preferred the set larger in number. These results can be interpreted as a preference for an individual distinct adult, versus multiple siblings. Our results support the hypothesis that size can draw a young chick’s attention towards adults before imprinting takes place.

When presented with large versus small yellow visual stimuli (Exp. 2), the chicks had no significant preference. A preference for the yellow and small stimuli observed towards the end of the experiment was not significant after correction for multiple comparisons. Should a preference for lighter and smaller objects be confirmed, it would suggest that chicks have a spontaneous preference for the association of adult (large and red) versus juvenile (small and yellow) features. Given the mounting evidence on cross-modal associations in chicks (Loconsole et al., 2021; Versace et al., 2024), this possibility should be further investigated. We hence compared the responses to red large versus red small stimuli (and yellow large vs. yellow small stimuli) in Experiment 3. As predicted, we found that the preference for red was stronger when the stimulus was a single large object compared to when the same area was presented as five small objects. This shows that the larger size enhances the preference for the red colour.

Comparing the two experiments, the yellow stimuli were consistently less attractive. Chicks approached red stimuli faster and spent more time with red stimuli overall, in line with previous studies (Regolin et al., 2011; Wang et al., 2024) (see also Supplementary Materials in S. Wang et al., 2024). When yellow stimuli were presented, chicks spent significantly less time in the areas close to the stimuli, and significantly fewer chicks approached the stimuli. A lower interest for the yellow stimuli has been previously observed in experiments that didn’t manipulate the size of visual stimuli (Kovach, 1971; Salzen et al., 1971; Schaefer & Hess, 1959). We recently showed that this yellow colour is not only less attractive but produces slower learning and generalisation. Further experiments should clarify to what extent the effect we have observed is driven by hue, brightness, a combination of colour features and the contrast with the background. Hens are dark compared to the background (McBride et al., 1969;Wang et al., 2024), which could also explain the attraction for dark objects outlined in previous studies (Versace et al., 2016)). Previous evidence on colour preferences in chicks is non-univocal, likely due to the fact that chicks have been tested with different methods, tasks, backgrounds, and at different ages (Gray, 1961; e.g., Ham & Osorio, 2007; Kovach, 1971; Salzen et al., 1971). However, our results are in line with the consensus that red and to a lesser extent blue are consistently attractive colours for chicks (Kovach, 1971; Salzen et al., 1971; Schaefer & Hess, 1959; Wang et al., 2024), while green and yellow elicit weaker approach responses (Ham & Osorio, 2007; Salzen et al., 1971). Interestingly, it has been found that chicks do imprint on yellow objects, but the process takes longer (Bateson & Jaeckel, 1976; Wang et al., 2024). The longer exposure required to imprint on yellow objects may enable chicks to imprint on both adult and siblings, thus keeping group cohesion, and at the same time prioritise the affiliative responses towards the adults. In natural settings, already few weeks after hatching chicks may remain in contact with the siblings but not with the hen (McBride et al., 1969).

This first work on predispositions for features that indicate the presence of visual adult features adds to the growing body of evidence on spontaneous predispositions. Such predispositions support the adaptive responses of animals at the start of life to aid predator avoidance (Hébert et al., 2019), preferential attention to animate objects (Bliss et al., 2023; Johnson & Horn, 1988; Lemaire & Vallortigara, 2023; Rosa-Salva et al., 2021; Versace & Martinho-Truswell, 2018) and imprinting (S. Wang et al., 2024) on care-giving adults, as discussed here.

## Supplementary Information

Below is the link to the electronic supplementary material.

Supplementary file1 (XLSX 37 KB)

Supplementary file2 (XLSX 34 KB)

Supplementary file3 (CSV 36 KB)

Supplementary file4 (CSV 31 KB)

Supplementary file5 (MP4 19710 KB)

Supplementary file6 (MP4 20227 KB)

## Data Availability

The data and materials for all experiments are available in the Online Supplementary Materials.

## Code availability

Example code is available on the public repository Zenodo: https://zenodo.org/records/10869154
